# Structure Characterization, Antioxidant and Immunomodulatory Activities of Polysaccharide from *Pteridium aquilinum* (L.) Kuhn

**DOI:** 10.3390/foods11131834

**Published:** 2022-06-22

**Authors:** Zhe-Han Zhao, Xian-Yan Ju, Kui-Wu Wang, Xin-Juan Chen, Hong-Xiang Sun, Ke-Jun Cheng

**Affiliations:** 1 School of Food Science and Biotechnology, Zhejiang Gongshang University, Hangzhou 310018, China; 13588665001@163.com; 2 Hangzhou Fuchun No.9 Primary School, Hangzhou 311400, China; xianyanju1988@163.com; 3 Institute of Vegetable, Zhejiang Academy of Agricultural Sciences, Hangzhou 310021, China; xjchenshanxi@126.com; 4 College of Animal Sciences, Zhejiang University, Hangzhou 310058, China; sunhx@zju.edu.cn; 5 Chemical Biology Center, Lishui Institute of Agriculture and Foresty Sciences, Lishui 323000, China; chengkejun2011@126.com

**Keywords:** *Pteridium aquilinum*, Pteridaceae, structure characterization, antioxidant activity, immunomodulatory activity

## Abstract

*Pteridium aquilinum* (L.) Kuhn (Pteridaceae family) has been widely used as a food and medicine in China and Korea. Previous studies indicate that *P. aquilinum* contains a variety of bioactive chemical components such as flavonoids, phenols, terpenoids, saponins, polysaccharides, and so on. In the present study, a novel polysaccharide (named as PAP-3) with average molecular weight of 2.14 × 10^5^ Da was obtained from *P. aquilinum*. The structure was studied through physicochemical and spectroscopic analysis. The results indicated that PAP-3 consists of arabinose, rhamnose, fucose, galactose, mannose, and xylose in a molar ratio of 1.58:1.00:3.26:4.57:4.81:3.33. The polysaccharide is mainly composed of (1→2)-linked xylose and (1→3,6)-linked mannose on the main chain, with (1→2)-linked xylose, (1→6)-linked mannose, and (1→6)- and (1→3,6)-linked galactose as side chains. Galactose, fucose, and xylose are located at the end of the side chains. The in vitro immunomodulatory and antioxidant activities were assayed. PAP-3 has strong free-radical scavenging activity on DPPH and ABTS radicals and significant immunomodulatory activity on RAW264.7 cells. These data provide useful information for further study on the polysaccharides of *P. aquilinum* and their applications in the food and medical industries.

## 1. Introduction

*Pteridium* is one of two genera in the plant Pteridaceae family. About 13 species are grown worldwide, but mainly in the tropics, and 6 of them are found in China. *Pteridium aquilinum* (L.) Kuhn is one of the most widely distributed ferns in the world. It has traditional application in ethnomedicines and as a food ingredient. The tender leaves and stems of this plant are used as vegetables in Korean and Chinese cuisines [1,2]. They are usually eaten after boiling because of ptaquiloside, an undesirable substance which is carcinogenic in some animals [3]. *P. aquilinum* is enriched with bioactive ingredients and has many health benefits, such as immunomodulatory, anti-carcinogenic, anti-bacterial, anti-inflammatory, and anti-oxidative properties [1,2,3,4,5].

Polysaccharides from botanical resources have received increasing interest from the world because of their novel structures and diverse bioactivities without harm on the growth of normal cells [6,7,8,9,10,11,12,13]. The bioactivities of polysaccharides are related to their physicochemical properties, including monosaccharide composition, molecular weight, glycosidic bonds, as well as their main-chain and side-chain conformations. The polysaccharides isolated from the Pteridaceae family have many good biological activities such as antioxidant and immune activity [14,15,16,17]. These articles focus on the activity of Pteridaceae polysaccharide but less on their structure. As part of our continuous research on active plant polysaccharides, here we report the extraction, isolation, and purification of a novel natural, water-soluble polysaccharide (PAP-3) from *P. aquilinum* using an anion-exchange (DEAE–Sepharose Fast Flow) and a Sepharose 4B column chromatography. In addition, the structural characteristics and the antioxidant and immunomodulatory activities of the purified polysaccharide are also investigated. The elucidation of the fine structure and the bioactivity assay in vitro of PAP-3 can provide more scientific information support for the previous conclusions on the structure of *Pteridium* polysaccharides. It is also helpful to understand the structure–function relationship of different fern polysaccharides and expand the application of fern resources.

## 2. Materials and Methods

### 2.1. Materials and Instruments

Monosaccharide standards (l-arabinose (l-Ara), l-rhamnose (l-Rha), d-fucose (d-Fuc), d-galactose (d-Gal), d-mannose (d-man), and d-xylose (d-Xyl)) and Dextran standards (T-670, T-270, T-150, T-50, T-10), trifluoroacetic acid (TFA), lipopolysaccharide (LPS), MTT, and HPLC-MeOH were obtained from Sigma Chemical Co., St. Louis, MI, USA. DEAE-Sepharose Fast Flow and Sepharose 4B were from GE Healthcare Bio-Sciences AB, Uppsala, Sweden. HPGPC analysis: Waters 2695 HPLC system, 2424 ELSD (Waters Corporation, Milford, MA, USA), Ultra hydrogel 2000 column. GC/GC–MS analysis: Agilent 7890A/5975C system (Agilent Technologies Inc., Santa Clara, CA, USA). NMR analysis: Bruker AVANCE III 500 spectrometer (Bruker Corporation, Rheinstetten, Germany) at 333 K (99.9% D_2_O). 

### 2.2. Polysaccharide Preparation from P. aquilinum

The aerial part of *P. aquilinum* sample was collected from Tianmu moutain, Hangzhou, China, and identified by Prof. Bin Wu (Zhejiang University, Hangzhou, China). The dried sample was crushed into 20 meshes and defatted by extracting twice with refluxing ethanol (95% *v*/*v*, 2.5 h per time). After removing the solvents, the residue was subjected to hot distilled water extraction (1:10, *v*/*v*, 95 °C, 2 h, 3 times). The extracts were combined and concentrated at 55 °C by rotary evaporator (Buchi R-210, BUCHI Labortechnik AG, Flawil, Switzerland) and then precipitated with 75% ethanol at 4 °C overnight. After centrifugation (6744× *g*, 5 min), the precipitate was re-dissolved in ultra-pure water to remove the protein by Sevage method (*n*-butanol: Chloroform, 1:4 *v*/*v*) three times. The solutions were centrifuged (6744× *g*, 5 min) and then dialyzed (MWCO 3500 Da, 48 h), vacuum concentrated (55 °C), and lyophilized to afford the crude polysaccharide (cPAP). 

cPAP was dissolved in ultra-pure water (30 mg/mL), filtered (0.45 μm) and fractionated by an anion-exchange chromatography column (CC, 5.0 i.d × 50 cm) using DEAE Sepharose Fast Flow, and eluted with ultra-pure water and 0.1, 0.3, and 0.5 M NaCl at 1 mL/min, successively. The carbohydrate content of the eluate was detected by the phenol-sulfuric acid assay method [18]. The 0.3 M sub-fraction was further chromatographed with a Sepharose 4B gel-permeation column, using ultra-pure water as eluent. A symmetrical sharp peak was collected, dialyzed to obtain the novel purified polysaccharide named PAP-3.

### 2.3. General Analysis of Purified Polysaccharide PAP-3

The homogeneity and average molecular weight (Mw) of PAP-3 were detected by HPGPC. Mobile phase: ultra-pure water. Flow rate: 0.35 mL/min. The Mw of PAP-3 was calculated by reference to the calibration curve made using Dextran standards. 

### 2.4. Determination of Monosaccharide Composition 

The sample of PAP-3 (5 mg) was completely hydrolyzed with TFA (1 mL, 2 M, 120 °C, 2.5 h) and reduced with hydroxylamine hydrochloride and pyridine (90 °C, 0.5 h). The inositol, obtained neutral sugars, and monosaccharide standards were acetylated and analyzed by GC utilizing the reported method [17]. The program of temperature for GC was: 170 °C (maintain 1 min), raised to 210 °C (5 °C/min), then raised to 260 °C (10 °C/min, maintain 1 min). Carrier gas: N_2_, 0.6 mL/min.

### 2.5. Partial Acid Hydrolysis

PAP-3 (20 mg) was partially hydrolyzed with TFA (1 mL, 0.05 M, 100 °C, 16 h) and dialyzed (MWCO 3500 Da, 48 h). Non-dialyzable sample (inside bag, namely PAP-3-I) and dialyzable fraction sample (outside bag, namely PAP-3-O) collected separately and evaporated to dryness at 55 °C. The monosaccharide compositions of PAP-3-I and PAP-3-O were detected by GC use the method as Section 2.4.

### 2.6. Periodate Oxidation and Smith Degradation

PAP-3 (20 mg) was oxidized with NaIO_4_ (0.015 M, 25 mL, 120 h). Ethylene glycol (1 mL) was used to destroy the excess NaIO_4_. The consumption of periodate was detected by UV spectroscopy (223 nm) [19]. The generated products of formic acid were titrated by NaOH (0.01 M). After being reduced by NaBH_4_ and neutralized with HOAc to pH 7.0, the solution was then hydrolyzed in TFA (2 M, 120 °C, 3 h) in order to analyze the monosaccharide compositions by GC.

### 2.7. Methylation Analysis

PAP-3 (20 mg) was methylated using the method reported before [19]. The methylated sample was extracted with CHCl_3_ and detected by IR spectroscopy (Nicolet iS 10 FT-IR Spectrometer, Thermo Scientific, USA). PAP-3 sample was ground with potassium bromide powder and pressed into pellets for FT-IR spectral measurement in the wavenumber range of 4000–400 cm^−1^. The pre-methylated product was hydrolyzed in TFA (2 M, 120 °C, 3 h), reduced by sodium borohydride, and then acetylated by the mixed solution of acetic anhydride/pyridine (1:1 *v*/*v*) to produce alditol acetates. The derivatives were detected by GC-MS. 

### 2.8. In vitro Antioxidant Activities Assay

#### 2.8.1. DPPH Free Radical Scavenging Activity

The scavenging ability of PAP-3 on DPPH free radicals was evaluated using the method reported with some modifications [20]. First, a series of PAP-3 solutions (50, 100, 200, 500, 1000, 2000 μg/mL) were prepared. Then, DPPH solution (2 mL, 0.1 mM) was added into 2 mL of above prepared solution separately, then blending. The mixture was placed in darkroom at 37 °C for 30 min and then measured at *λ*_517_ nm using Synergy H1 multi-mode reader (BioTek, Winusky, VT, USA).

Scavenging activity (%) = [1 − (*A*_1_ − *A*_2_)/*A*_0_] × 100%
*A*_0_: Absorption (Abs) of the DPPH solution without PAP-3;
*A*_1_: Abs of the reaction mixture;
*A*_2_: Abs of PAP-3 without the DPPH solution.

#### 2.8.2. ABTS Free Radical Scavenging Activity

ABTS free radical scavenging ability of PAP-3 was determined in terms of the method conducted before with some modifications [21]. ABTS radical cations were prepared by mixing 7 mM ABTS/2.45 mM potassium persulphate (1:1, *v*/*v*) and incubated in darkroom for 16 h. Then, the solution was diluted using phosphate buffer (10 mM, pH 7.4) to an UV absorbance of 0.70 ± 0.02 at *λ*_734_ nm. PAP-3 solution with various concentrations (50–2000 μg/mL, 2 mL) was added to cationic ABTS radical solution (2 mL), shaken and incubated (37 °C, 30 min), and measured at *λ*_734_ nm using Synergy H1 multi-mode reader.

Scavenging activity (%) = [1 − (*A*_1_ − *A*_2_)/*A*_0_] × 100%
*A*_0_: Abs of the ABTS solution without PAP-3;
*A*_1_: Abs of the reaction mixture;
*A*_2_: Abs of PAP-3 without the ABTS solution.

### 2.9. In Vitro Immunomodulatory Activity Assay of PAP-3

The immunomodulatory effect of PAP-3 on RAW264.7 cells was assayed using the method reported before [22]. Briefly, the effect of PAP-3 on the viability of RAW264.7 cells was determined by MTT method. RAW264.7 cells were seeded in a 96-well plate and incubated at 37 °C in a humidified atmosphere with 5% CO_2_ for 24 h. Then, the various concentrations of PAP-3 (0–200 μg/mL) were added into each well and incubated at 37 °C for 24 h. Four hours prior to the end of incubation, 50 μL of MTT solution (2000 μg/mL) was added to each well. The plates were further incubated for 4 h and then centrifuged (1400× *g*, 5 min). The untransformed MTT was removed carefully by pipetting. To each well, 150 μL of a DMSO solution was added, and the absorbance was evaluated in an ELISA reader at 570 nm with a 630 nm reference. 

RAW264.7 cells were cultured with different concentrations of PAP-3 (0–200 μg/mL) for 24 h then the nitrite contents were determined by Griess reaction. Briefly, 100 μL aliquots of the supernatant and equal volumes of the Griess reaction solutions were distributed in a 96-well plate. After reacting for 10 min at room temperature, the absorbance was recorded in an ELISA reader at 540 nm, and the concentrations of NO_2_^−^ were determined from a sodium nitrite standard curve.

## 3. Results and Discussion

### 3.1. Extraction, Purification, and Molecular Weight of PAP-3

**Figure 1:**
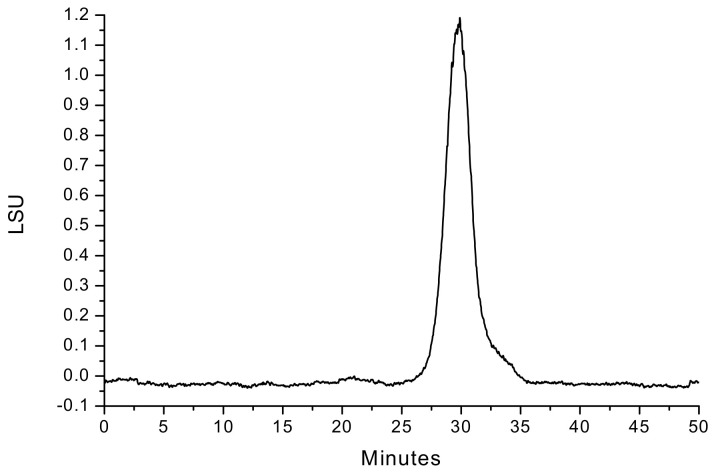
The HPGPC chromatogram of PAP-3.

The crude polysaccharide (cPAP, yield 14.02%) was obtained from *P. aquilinum* by hot water extraction, ethanol precipitation, and deproteinization. The purification of cPAP on the DEAE Sepharose Fast Flow CC (Figure S1) and Sepharose 4B CC led to a signal and symmetrical sharp peak (Figure S2). The homogeneity of purified polysaccharide (PAP-3) was confirmed by HPGPC (Figure 1), and its average molecular weight was 2.14 × 10^5^ Da, which was calculated using the Standard curve of Dextrans (Figure S3). The weak UV absorption peak at 260–280 nm suggests that the protein and/or nucleic acid content in PAP-3 is very low (Figure S4). 

### 3.2. Structural Characterization of PAP-3

The structure of PAP-3 was characterized using a combination of physicochemical (complete/partial acid hydrolysis, periodate oxidation–Smith degradation, and methylation analysis), chromatographic, and spectroscopic (GC, GC-MS, IR, and NMR) methods.

**Figure 2:**
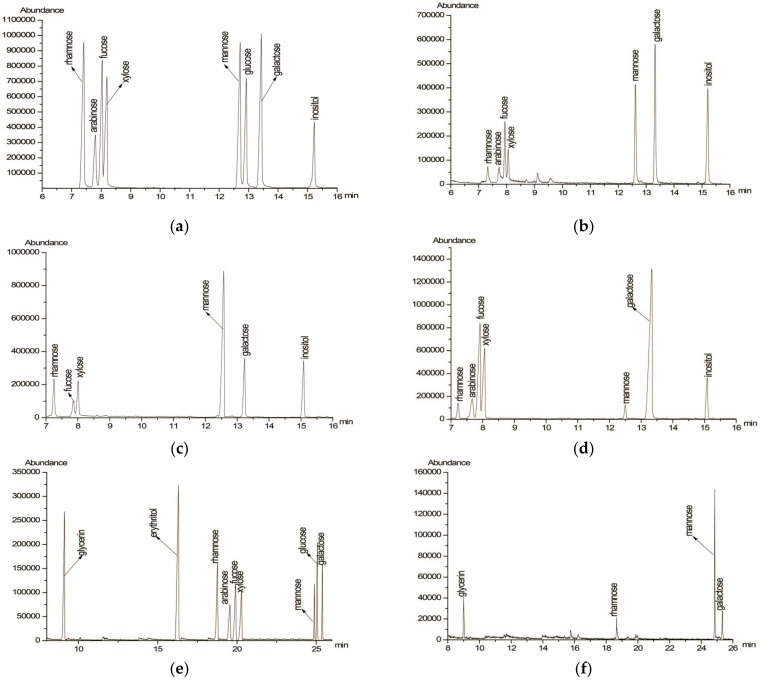
GC chromatograms of standard monosaccharides (**a**); monosaccharide compositions of PAP-3 (**b**); monosaccharide compositions of PAP-3-I (**c**); monosaccharide compositions of PAP-3-O (**d**); standard monosaccharides, glycerol and erythritol (**e**); products of Smith degradation of PAP-3 (**f**).

Firstly, PAP-3 was completely acid hydrolyzed. The GC chromatogram of the complete hydrolysis products of PAP-3 (Figure 2b) showed the presence of alditol acetates of l-arabinose (l-Ara), l-rhamnose (l-Rha), d-fucose (d-Fuc), d-galactose (d-Gal), d-mannose (d-man), and d-xylose (d-Xyl) in a molar ratio of 1.58:1.00:3.26:4.57:4.81:3.33 (Table 1), which was confirmed by the retention time of the standard monosaccharide derivatives (Figure 2a). Xu et al. [16] and Song et al. [17] also reported polysaccharides isolated from *P. aquilinum*. The monosaccharide composition of these polysaccharides is different from that of PAP-3. Thus, PAP-3 is a novel natural polysaccharide isolated from *P. aquilinum.*

PAP-3 was then partially acid hydrolyzed, and the sugar compositions of PAP-3-I (Figure 2c) and PAP-3-O (Figure 2d) were analyzed separately. The results showed (Table 1) that PAP-3-I contained l-Rha, d-Fuc, d-Gal, d-Man, and d-Xyl in a molar proportion of 2.25:1.00: 1.87:8.92:4.02, while PAP-3-O was composed of l-Ara, l-Rha, d-Fuc, d-Gal, d-Man, and d-Xyl in a molar ratio of 7.53:1.66:12.62:13.48:1.00:15.66, suggesting that d-Man was mostly present in the main chain while l-Ara, d-Fuc, and d-Gal were mainly present in the side chain, and d-Xyl was both in the main chain and the side chain. Thus, the main backbone of PAP-3 consisted of d-Xyl and d-Man (1.0:2.2).

PAP-3 was oxidized with NaIO_4_ for 120 h. Each mole of sugar residue for PAP-3 consumed 1.14 mol of NaIO_4_ and generated 0.39 mol of HCOOH. This indicated that the (1→6)-linked glycosyl bonds and/or non-reducing terminal residues amounted to 39% in PAP-3, with (1→2)-/(1→4)-/(1→4,6)-/(1→2,6)-linked bonds and (1→3)-/(1→2,3)-/(1→2,4)-/(1→3,4)-/(1→3,6)-/(1→2,3,4)-linked bonds amounting to 36% and 25%, respectively. The GC graph (Figure 2e,f) of the Smith degradation products showed the presence of rhamnose, galactose, and mannose (Table 1), indicating that residues of these glycosyls were non-oxidizable, such as (1→2,3)-/(1→2,4)-/(1→2,3,4)-/(1→3)-/(1→3,4)-/(1→3,6)-linked. The absence of arabinose, fucose, and xylose demonstrated that these glycosyl residues were (1→)-/(1→2)-/(1→2,6)-/(1→4)-/(1→4,6)-/(1→6)-linkages, which can be oxidized. The presence of glycerol indicated that these residues were (1→)-/(1→2)-/(1→2,6)-/(1→6)-linked. Additionally, the lack of detected erythritol indicates that there are no (1→4)-/(1→4,6)-linked residues [23].

**Figure 3:**
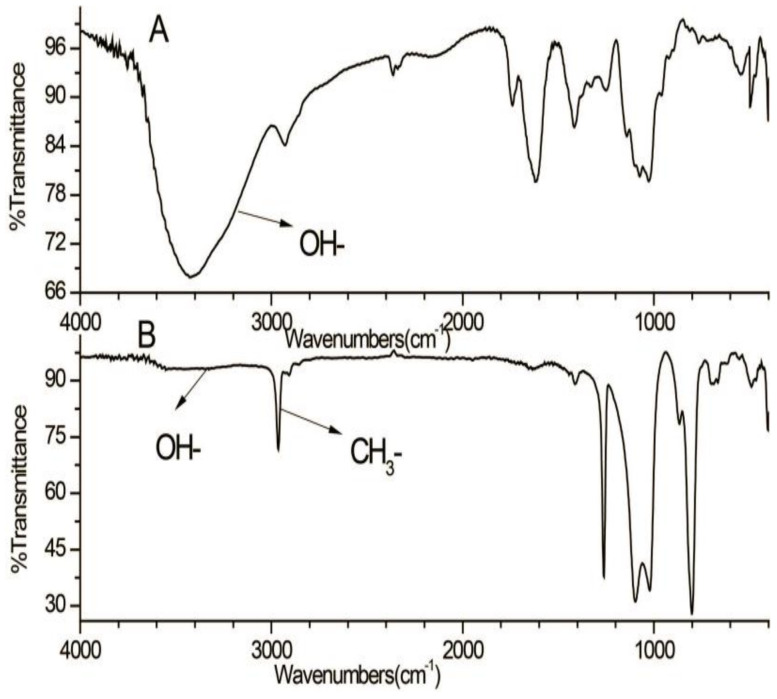
IR spectrum of PAP-3 (**A**) and its methylated product (**B**).

**Figure 4:**
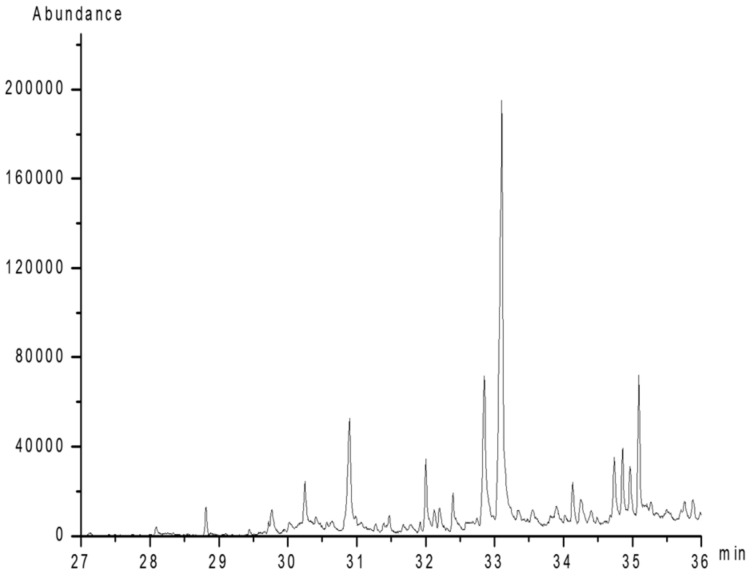
Total ionic chromatogram of methylated PAP-3.

As shown in Figure 3, the disappearance of the OH vibration absorption peak (3100–3600 cm^−1^) indicated the complete methylation of the free hydroxyls. After complete acid hydrolysis and alditol acetylation of the per-methylated polysaccharide, the obtained partially methylated alditol acetates were analyzed by GC–MS. The results showed eight types of main residues (Figure 4), which were identified as 2,3,4,6-Me_4_-Gal*p* (A), 2,3,4-Me_3_-Gal*p* (B), 2,4-Me_2_-Gal*p* (C), 2,3,4-Me_3_-Fuc*p* (D), 2,3,4-Me_3_-Man*p* (E), 2,4-Me_2_-Man*p* (F), 2,3,4-Me_3_-Xyl*p* (G), and 3,4-Me_2_-Xyl*p* (H) with the relative molar proportion of 1.0:1.3:2.1:2.2:1.5:2.5:1.2:3.2 (Table 2). 

These data suggest that PAP-3 contains (1→2)-linked d-Xyl and (1→3,6)-linked d-Man (1.0:2.2) on the main chain, with (1 → 2)-linked d-Xyl, (1→6)-linked d-Man, (1→6)- and (1→3,6)-linked d-Gal as side chains. d-Fuc, d-Gal, and d-Xyl are located at the end of the polysaccharide side chains. 

**Figure 5:**
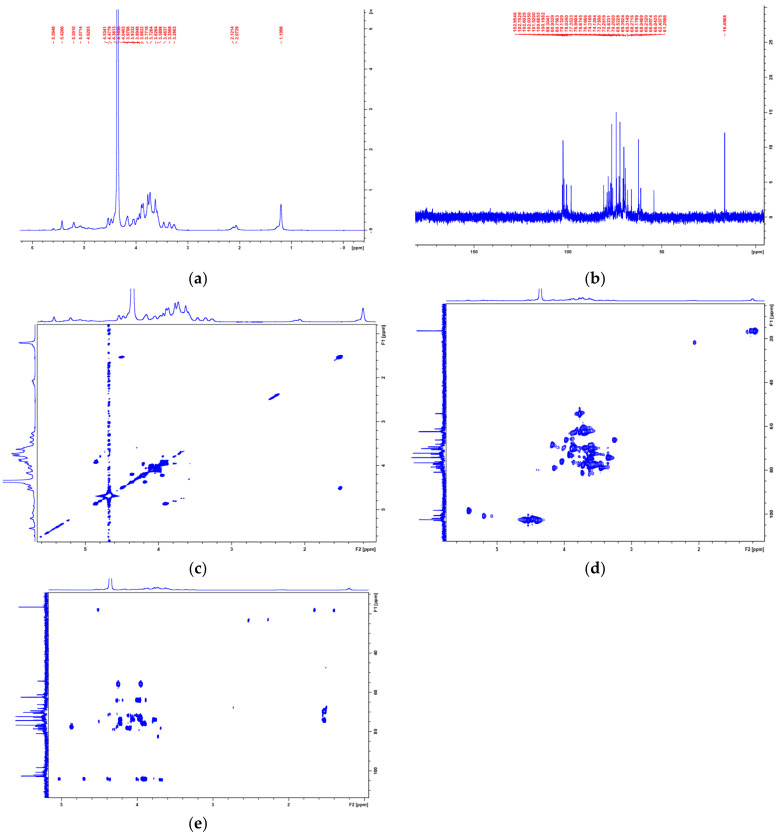
NMR spectra of PAP-3. ((**a**): ^1^H NMR; (**b**): ^13^C NMR; (**c**): ^1^H/^1^H COSY; (**d**): HSQC; (**e**): HMBC).

**Figure 6:**
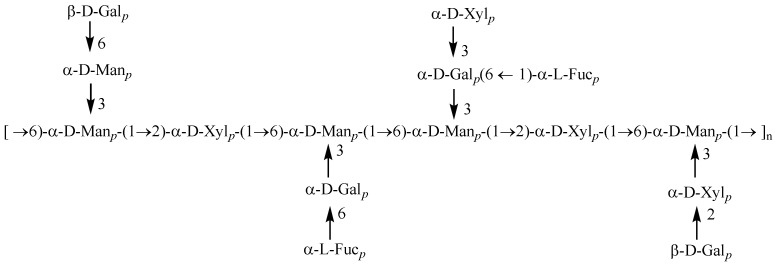
Possible structures of PAP-3 from *P. aquilinum*.

The structure of PAP-3 was further characterized based on the NMR spectra data (^1^H, ^13^C NMR, COSY, HSQC, and HMBC spectra) (Figure 5). The configuration of glycosidic bond (*α*- or *β*-) was confirmed by the chemical shifts in anomeric H and C atoms. Generally, the anomeric proton signals with chemical shifts ranging from δ_H_ 4.9 to 5.9 ppm or from 4.3 to 4.9 ppm indicate the existence of *α*- or *β*-configuration glycosidic bonds, respectively. Correspondingly, the ^13^C chemical shifts in the *α*- or *β*-configuration bonds often experience present at δ_C_ 97–102 or 102–107 ppm, respectively [9,11,24,25,26]. The signals of H1/C1 at δ_H_/δ_C_ 4.53/102.60, 4.48/102.04, and 4.42/102.95 (Table 3) confirmed the existence of →3,6)-β-d-Gal*p*-(1→ [27,28], T-β-d-Gal*p*-(1→ [27,29], and → 6)-β-d-Gal*p*-(1→ [27,30], respectively. The signals (H1/C1) at δ5.42/98.30, 5.20/100.69, 5.07/100.20, 4.92/101.52, and 4.90/102.75 showed the presence of →3,6)-α-d-Man*p*-(1→ [26,30], →2)-α-d-Xyl*p*-(1→ [31], → 6)-α-d-Man*p*-(1 → [32,33], T-α-l-Fuc*p*-(1→ [34,35], and T-α-d-Xyl*p*-(1→ [31], respectively. The 3,6-O-substituted α-d-Man*p* and 3,6-O-substituted *β*-d-Gal*p* units were confirmed by C-3/C-6 chemical shift in δ_C_ 80.75/66.10 and 80.96/66.23 ppm, respectively, while the 6-O-substituted *β*-d-Man*p* and *β*-d-Gal*p* units were downfield to δ_C_ 66.09/66.04 (C-6) ppm, respectively. Signals relative to the (1→2)-linked α-d-Xyl*p* were observed at δ_C_ 79.13 ppm (C-2). The signals in the upfield at *δ*_H_/*δ*_C_ 1.20/16.5 ppm were assigned to fucose. The NMR data of the eight main monosaccharide residues are listed in Table 3, and the predicted structure of PAP-3 is shown in Figure 6.

### 3.3. Antioxidant Activities Analysis

#### 3.3.1. DPPH Free Radical Scavenging Activity of PAP-3

**Figure 7:**
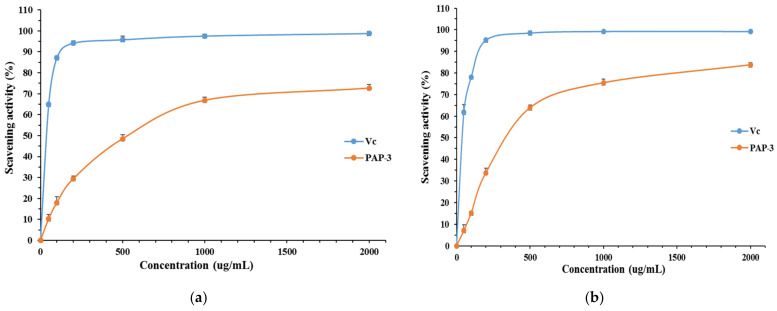
Scavenging activity of DPPH (**a**), ABTS (**b**) free radicals of PAP-3 from *P. aquilinum*.

DPPH radicals are widely used to evaluate the free radical scavenging ability of natural compounds. The polysaccharide showed preferable scavenging ability against DPPH free radicals (Figure 7a). The scavenging power elevated from 10.22% to 72.64% according the concentration of PAP-3 increased from 50 to 2000 μg/mL. As a control, Vc displayed higher scavenging activity than PAP-3, reaching 98.79% at 2000 μg/mL. Previous studies have shown that ferns have good antioxidant activity, but these studies mainly involve small molecular compounds or crude extracts [1,36]. Xu et al. [16] reported a water-soluble polysaccharide (PLP), obtained from fern *P. aquilinum*, showed 50.3% scavenging effects on DPPH radicals at a dose of 800 μg/mL. Wang et al. reported that a *Pteridium aquilinum*-derived oligosaccharide (PAO) showed high hydroxyl radical scavenging activity (82%) at the concentration of 80 μg/mL [2]. The activity of PAP-3 is slightly better than that of PLP.

#### 3.3.2. ABTS Free Radical Scavenging Activity of PAP-3 

ABTS radical decolorization assay is often applied to evaluate the total antioxidant power of plant polysaccharides [37,38]. Figure 7b showed the scavenging rate of PAP-3 against ABTS free radicals. PAP-3 exhibited increasing abilities at the concentration range of 50 to 2000 μg/mL. The ABTS radical scavenging ability of PAP-3 was 83.73% at 2000 μg/mL, suggesting that PAP-3 was a potential ABTS radical-scavenger. 

### 3.4. In Vitro Immunomodulatory Activity of PAP-3 

**Figure 8:**
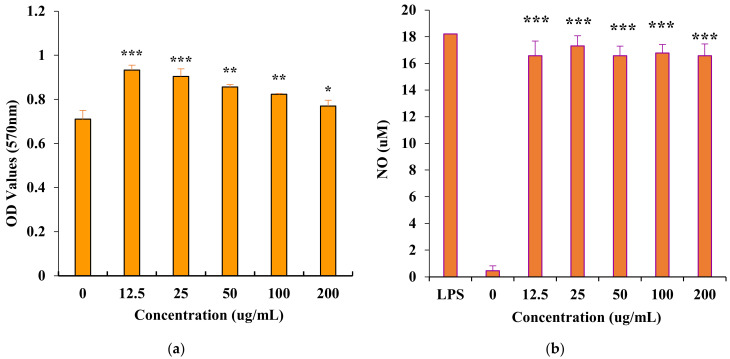
Effects of PAP-3 on the proliferation of RAW264.7 cells (**a**) and on nitric oxide (NO) production in RAW264.7 cells (**b**). (The values are presented as means ± SD (*n* = 4). Significant differences were designated as * *p* < 0.05, ** *p* < 0.01, *** *p* < 0.001. LPS: Positive Control).

Immunostimulation is regarded as one of the important body’s defense strategies for preventing and fighting many diseases. Plant-derived polysaccharides have attracted impressive attention and play important roles in immune system due to their structural diversity and low toxicity [7]. The immunomodulatory effect of PAP-3 on macrophage RAW264.7 cells was assayed within a concentration range of 0 to 200 μg/mL (Figure 8a). PAP-3 can induce the proliferation of macrophage RAW264.7 cells at 12.5 to 200 μg/mL. Moreover, no cytotoxic effects were detected up to 200 μg/mL. As shown in Figure 8b, incubating with PAP-3 can greatly induce NO product expression from RAW264.7 cells. The concentration of NO reached 17.31 μM after treatment with 25 μg/mL of PAP-3, and it is almost the same as that of the positive control (LPS, 10.0 μg/mL). The immune activity is almost consistent with that reported in the literature [17]. Therefore, PAP-3 may play an important role in the host defense system.

## 4. Conclusions

This study reported the extraction, purification and identification of a hetero-polysaccharide PAP-3 from *P. aquilinum*. The spectroscopy and physicochemical property analyses indicated that PAP-3 is a novel natural polysaccharide. PAP-3 consists of (1→2)-linked d-Xyl and (1→3,6)-linked d-Man on the main chain and (1→2)-linked d-Xyl, (1→6)-linked d-Man, and (1→6)- and (1→3,6)-linked d-Gal as side chains. Galactose, fucose, and xylose are located at the termini of the side chains. The results of anti-oxidant and immunomodulatory assays showed that PAP-3 has strong scavenging activity on DPPH and ABTS free radicals and could promote the proliferation and NO production of RAW264.7 cells, showing that PAP-3 has potential application value as a functional food ingredient.

## Figures and Tables

**Table 1:** Results of monosaccharide composition, partial acid hydrolysis, Smith degradation of PAP-3 from *P. aquilinum*.

	Gly	Ery	L-Ara	L-Rha	D-Fuc	D-Gal	D-Man	D-Xyl
PAP-3	/	/	1.58	1.00	3.26	4.57	4.81	3.33
PAP-3-I	/	/	-	2.25	1.00	1.87	8.92	4.02
PAP-3-O	/	/	7.53	1.66	12.62	13.48	1.00	15.66
Smith degradation	+	-	-	+	-	+	+	-

Gly: Glycerol; Ery: Erythritol; +: Detected; -: Not detected; /: No testing required.

**Table 2:** Results of the methylation of PAP-3 from *P. aquilinum*.

	Methylated Sugar	Deduced Linkage Patten	Major Mass Ion Fragmentation	Relative Molar Ratio
A	2,3,4,6-Me_4_-Gal*p*	1-Linked Gal*p*	87, 101, 117, 129, 145, 161, 205	1.0
B	2,3,4-Me_3_-Gal*p*	1,6-Linked Gal*p*	87, 101, 117, 129, 145, 161, 187, 205	1.3
C	2,4-Me_2_-Gal*p*	1,3,6-Linked Gal*p*	87, 99, 101, 129, 149, 161, 233	2.1
D	2,3,4-Me_3_-Fuc*p*	1-Linked Fuc*p*	81, 101, 117, 126, 155, 207, 233	2.2
E	2,3,4-Me_3_-Man*p*	1,6-Linked Man*p*	89, 101, 117, 129, 145, 161, 173, 205	1.5
F	2,4-Me_2_-Man*p*	1,3,6-Linked Man*p*	87, 99, 117, 129, 173, 189, 207, 233	2.5
G	2,3,4-Me_3_-Xyl*p*	1-Linked Xyl*p*	71, 87, 101, 117, 129, 161	1.2
H	3,4-Me_2_-Xyl*p*	1,2-Linked Xyl*p*	87, 101, 129, 145, 161, 189	3.2

**Table 3:** ^1^H and ^13^C NMR Chemical Shifts of PAP-3 from *P. aquilinum*.

	Deduced Linkage	NMR Data (δ in ppm)					
		H1/C1	H2/C2	H3/C3	H4/C4	H5/C5	H6/C6
A	T-β-d-Gal*p*-(1→	4.48/102.04	3.36/74.12	3.59/74.32	3.70/69.39	3.58/77.35	3.72/61.21
B	→ 6)-β-d-Gal*p*-(1→	4.42/102.95	3.46/72.75	3.36/74.12	3.89/70.63	4.05/76.17	3.27, 3.97/66.04
C	→3,6)-β-d-Gal*p*-(1→	4.53/102.60	3.63/74.32	3.73/80.95	4.02/69.52	3.73/76.67	3.27, 3.97/66.23
D	T-α-l-Fuc*p*-(1→	4.92/101.52	3.71/69.39	3.90/69.52	3.65/74.32	4.18/68.05	1.20/16.50
E	→6)-α-d-Man*p*-(1→	5.07/100.20	3.67/74.32	3.83/78.02	3.73/69.32	3.59/72.28	3.27, 3.97/66.09
F	→3,6)-α-d-Man*p*-(1→	5.42/98.30	3.69/72.28	3.75/80.75	3.67/74.32	3.58/72.28	3.27, 3.97/66.10
G	T-α-d-Xyl*p*-(1→	4.90/102.75	3.59/74.32	3.86/76.96	3.62/72.28	3.77, 3.86/62.46	
H	→2)-α-d-Xyl*p*-(1→	5.20/100.69	4.16/79.13	3.59/74.32	3.58/72.28	3.77, 3.86/62.46	

## Data Availability

Data is contained within the article or Supplementary Materials.

## Supplementary Materials

The following supporting information can be downloaded at: https://www.mdpi.com/article/10.3390/foods11131834/s1, Figure S1: Purification graph of polysaccharides on DEAE Sepharose Fast Flow column chromatography; Figure S2: Purification graph of PAP-3 on Sepharose 4B column chromatography; Figure S3: Standard curve of Dextrans; Figure S4: The UV spectrum of PAP-3.

## References

[1] Kardong D., Upadhyaya S., Saikia L.R. (2013). Screening of phytochemicals, antioxidant and antibacterial activity of crude extract of *Pteridium aquilinum* Kuhn. J. Pharm. Res..

[2] Wang H., Wu H. (2013). Preparation and antioxidant activity of *Pteridium aquilinum*-derived oligosaccharide. Int. J. Biol. Macromol..

[3] Castillo U.F., Ojika M., Alonso-Amelot M., Sakagami Y. (1998). Ptaquiloside Z, a New Toxic Unstable Sesquiterpene Glucoside from the Neotropical Bracken Fern *Pteridium aquilinum* Var. Caudatum. Bioorg. Med. Chem..

[4] Fletcher M.T., Brock I.J., Reichmann K.G., McKenzie R.A., Blaney B.J. (2011). Norsesquiterpene Glycosides in Bracken Ferns (*Pteridium esculentum* and *Pteridium aquilinum* subsp. wightianum) from Eastern Australia: Reassessed Poisoning Risk to Animals. J. Agric. Food Chem..

[5] Castillo U.F., Wilkins A.L., Lauren D.R., Smith B.L., Alonso-Amelot M. (2003). Pteroside A2-a New Illudane-Type Sesquiterpene Glucoside from *Pteridium caudatum* L. Maxon, and the Spectrometric Characterization of Caudatodienone. J. Agric. Food Chem..

[6] Liu Q., Ma R., Li S., Fei Y., Lei J., Li R., Pan Y., Liu S., Wang L. (2022). Dietary Supplementation of *Auricularia auricula-judae* Polysaccharides Alleviate Nutritional Obesity in Mice via Regulating Inflammatory Response and Lipid Metabolism. Foods.

[7] Yu Y., Shen M., Song Q., Xie J. (2018). Biological activities and pharmaceutical applications of polysaccharide from natural resources: A review. Carbohydr. Polym..

[8] Qi Y., Wang L., You Y., Sun X., Wen C., Fu Y., Song S. (2022). Preparation of Low-Molecular-Weight Fucoidan with Anticoagulant Activity by Photocatalytic Degradation Method. Foods.

[9] Pan L.C., Zhu Y.M., Zhu Z.Y., Xue W., Liu C.Y., Sun H.Q., Yin Y. (2020). Chemical structure and effects of antioxidation and against α-glucosidase of natural polysaccharide from *Glycyrrhiza inflata* Batalin. Int. J. Biol. Macromol..

[10] Li S., Huo X., Qi Y., Ren D., Li Z., Qu D., Sun Y. (2022). The Protective Effects of Ginseng Polysaccharides and Their Effective Subfraction against Dextran Sodium Sulfate-Induced Colitis. Foods.

[11] Zhang Y., Zhou T., Wang H., Cui Z., Cheng F., Wang K. (2016). Structural characterization and in vitro antitumor activity of an acidic polysaccharide from *Angelica sinensis* (Oliv.) Diels. Carbohydr. Polym..

[12] Samtiya M., Aluko R.E., Dhewa T., Moreno-Rojas J.M. (2021). Potential Health Benefits of Plant Food-Derived Bioactive Components: An Overview. Foods.

[13] Jiang L.L., Gong X., Ji M.Y., Wang C.C., Wang J.H., Li M.H. (2020). Bioactive Compounds from Plant-Based Functional Foods: A Promising Choice for the Prevention and Management of Hyperuricemia. Foods.

[14] Silva G.B., Ionashiro M., Carrara T.B., Crivellari A.C., Tiné M.A.S., Prado J., Carpita N.C., Buckeridge M.S. (2011). Cell wall polysaccharides from fern leaves: Evidence for a mannan-rich Type III cell wall in *Adiantum raddianum*. Phytochemistry.

[15] Wu M.J., Weng C.Y., Wang L., Lianm T.W. (2005). Immunomodulatory mechanism of the aqueous extract of sword brake fern (*Pteris ensiformis* Burm.). J. Ethnopharmacol..

[16] Xu W., Zhang F., Luo Y., Ma L., Kou X., Huang K. (2009). Antioxidant activity of a water-soluble polysaccharide purified from *Pteridium aquilinum*. Carbohydr. Res..

[17] Song G.L., Wang K.W., Zhang H., Sun H.X., Wu B., Ju X.Y. (2017). Structural characterization and immunomodulatory activity of a novel polysaccharide from *Pteridium aquilinum*. Int. J. Biol. Macromol..

[18] Yu X.H., Liu Z.Y., Yang J.S., Yang X.Y., Wan R.L. (2010). Controlling Quality of *Astragalus* polysaccharide Meal by Combined TLC and Phenol-Sulfuric Acid Method. Med. Plant.

[19] Zhang H., Ye L., Wang K.W. (2010). Structural characterization and anti-inflammatory activity of two water-soluble polysaccharides from *Bellamya purificata*. Carbohydr. Polym..

[20] Hu J., Gao J., Zhao Z., Yang X. (2021). Response surface optimization of polysaccharide extraction from *Galla Chinensis* and determination of its antioxidant activity *in vitro*. Food Sci. Technol..

[21] Chen L., Huang G. (2019). Antioxidant activities of phosphorylated pumpkin polysaccharide. Int. J. Biol. Macromol..

[22] Sun H.X., Zhang J., Chen F.Y., Chen X.F., Zhou Z.H., Wang H. (2015). Activation of RAW264.7 macrophages by the polysaccharide from the roots of *Actinidia eriantha* and its molecular mechanisms. Carbohydr. Polym..

[23] Yin Y., Yu R., Yang W., Yuan F., Yan C., Song L. (2010). Structural characterization and anti-tumor activity of a novel heteropolysaccharide isolated from *Taxus yunnanensis*. Carbohydr. Polym..

[24] Tu J., Liu H., Wen Y., Chen P., Liu Z. (2021). A novel polysaccharide from *Hericium erinaceus*: Preparation, structural characteristics, thermal stabilities, and antioxidant activities in vitro. J. Food Biochem..

[25] Heiss C., Burtnick M.N., Roberts R.A., Black I., Azadi P., Brett P.J. (2013). Revised structures for the predominant O-polysaccharides expressed by *Burkholderia pseudomallei* and *Burkholderia mallei*. Carbohydr. Res..

[26] Yao H.Y.Y., Wang J.Q., Yin J.Y., Nie S.P., Xie M.Y. (2021). A review of NMR analysis in polysaccharide structure and conformation: Progress, challenge and perspective. Food Res. Int..

[27] Carlotto J., De Almeida Veiga A., De Souza L.M., Cipriani T.R. (2020). Polysaccharide fractions from *Handroanthus heptaphyllus* and *Handroanthus albus* barks: Structural characterization and cytotoxic activity. Int. J. Biol. Macromol..

[28] Zeng F., Chen W., He P., Zhan Q., Wang Q., Wu H., Zhang M. (2020). Structural characterization of polysaccharides with potential antioxidant and immunomodulatory activities from Chinese water chestnut peels. Carbohydr. Polym..

[29] Chen L., Liu J., Zhang Y., Dai B., An Y., Yu L. (2015). Structural, Thermal, and Anti-inflammatory Properties of a Novel Pectic Polysaccharide from Alfalfa (*Medicago sativa* L.) Stem. J. Agric. Food Chem..

[30] Chen J., Li L., Zhang X., Wan L., Zheng Q., Xu D., Li Y., Liang Y., Chen M., Li B. (2021). Structural characterization of polysaccharide from *Centipeda minima* and its hypoglycemic activity through alleviating insulin resistance of hepatic HepG2 cells. J. Funct. Foods.

[31] Makarova E.N., Shakhmatov E.G. (2021). Characterization of pectin-xylan-glucan-arabinogalactan proteins complex from Siberian fir *Abies sibirica* Ledeb. Carbohydr. Polym..

[32] San-Blas G., Prieto A., Bernabe M., Ahrazem O., Moreno B., Leal J.A. (2005). α-gal*f* 1→6-α-mannopyranoside side chains in *Paracoccidioides brasiliensis* cell wall are shared by members of the Onygenales, but not by galactomannans of other fungal genera. Med. Mycol..

[33] Smiderle F.R., Sassaki G.L., Van Griensven L.J.L.D., Iacomini M. (2013). Isolation and chemical characterization of a glucogalactomannan of the medicinal mushroom *Cordyceps militaris*. Carbohydr. Polym..

[34] Wang Y.X., Yin J.Y., Zhang T., Xin Y., Huang X.J., Nie S.P. (2021). Utilizing relative ordered structure theory to guide polysaccharide purification for structural characterization. Food Hydrocoll..

[35] Sigida E.N., Fedonenko Y.P., Shashkov A.S., Zdorovenko E.L., Konnova S.A., Ignatov V.V., Knirel Y.A. (2013). Structural studies of the O-specific polysaccharide(s) from the lipopolysaccharide of *Azospirillum brasilense* type strain Sp7. Carbohydr. Res..

[36] Tsumbu C.N., Deby-Dupont G., TitsZ M., Angenot L., Frederich M., Kohnen S., Mouithys-Mickalad A., Serteyn D., Franck T. (2012). Polyphenol Content and Modulatory Activities of Some Tropical Dietary Plant Extracts on the Oxidant Activities of Neutrophils and Myeloperoxidase. Int. J. Mol. Sci..

[37] Fan Y., He X., Zhou S., Luo A., He T., Chun Z. (2009). Composition analysis and antioxidant activity of polysaccharide from *Den drobium denneanum*. Int. J. Biol. Macromol..

[38] Luo A., Ge Z., Fan Y., Luo A., Chun Z., He X. (2011). In Vitro and In Vivo Antioxidant Activity of a Water-Soluble Polysaccharide from *Dendrobium denneanum*. Molecules.

