# Supplementary material for: Structure Characterization, Antioxidant and Immunomodulatory Activities of Polysaccharide from Pteridium aquilinum (L.) Kuhn
Source: Foods. 2022 Jun 22;11(13):1834. doi: 10.3390/foods11131834 (PMC9265270; doi:10.3390/foods11131834)
Supplement: Supplementary file 1 [file foods-11-01834-s001.zip › foods-1777210-supplementary.pdf]

Article

Structure Characterization, Antioxidant and Immunomodulatory Activities of Polysaccharide from *Pteridium aquilinum* (L.) Kuhn

Zhe-Han Zhao [1], Xian-Yan Ju [2], Kui-Wu Wang [1,*], Xin-Juan Chen [3], Hong-Xiang Sun [4] and Ke-Jun Cheng [5]

[1] School of Food Science and Biotechnology, Zhejiang Gongshang University, Hangzhou 310018, China; 13588665001@163.com
[2] Hangzhou Fuchun No.9 Primary School, Hangzhou 311400, China; xianyanju1988@163.com
[3] Institute of Vegetable, Zhejiang Academy of Agricultural Sciences, Hangzhou 310021, China; xjchenshanxi@126.com
[4] College of Animal Sciences, Zhejiang University, Hangzhou 310058, China; sunhx@zju.edu.cn
[5] Chemical Biology Center, Lishui Institute of Agriculture and Foresty Sciences, Lishui 323000, China; chengkejun2011@126.com
* Correspondence: wkwnpc@zjgsu.edu.cn; Tel.: +86-571-2800-8975

Supplementary Material

Figure S1. Purification graph of polysaccharides on DEAE Sepharose Fast Flow column chromatography
Figure S2. Purification graph of PAP-3 on Sepharose 4B column chromatography
Figure S3. Standard curve of Dextrans.
Figure S4. The UV spectrum of PAP-3

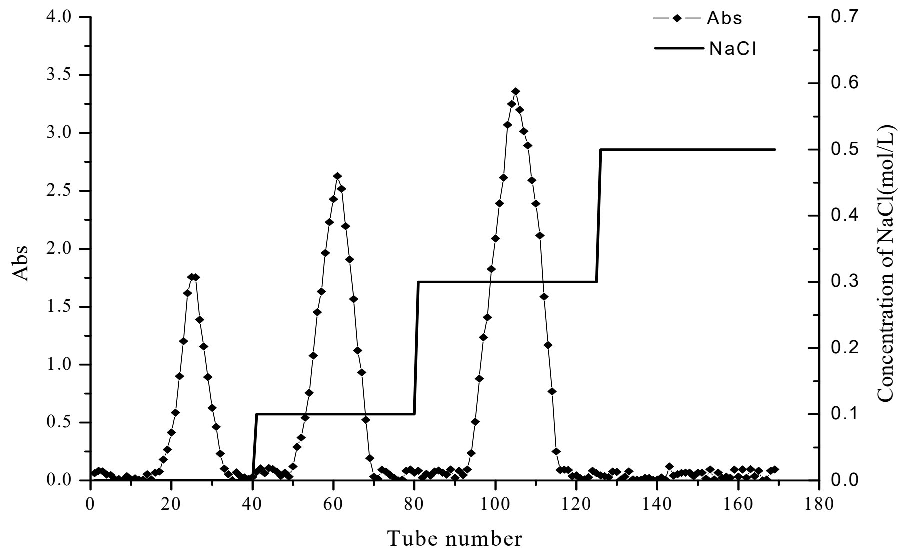

Figure S1. Purification graph of polysaccharides on DEAE Sepharose Fast Flow column chromatography

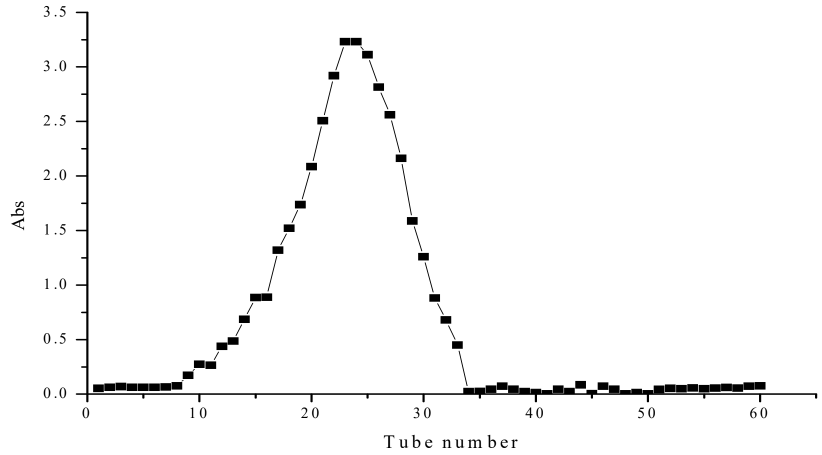

Figure S2. Purification graph of PAP-3 on Sepharose 4B column chromatography

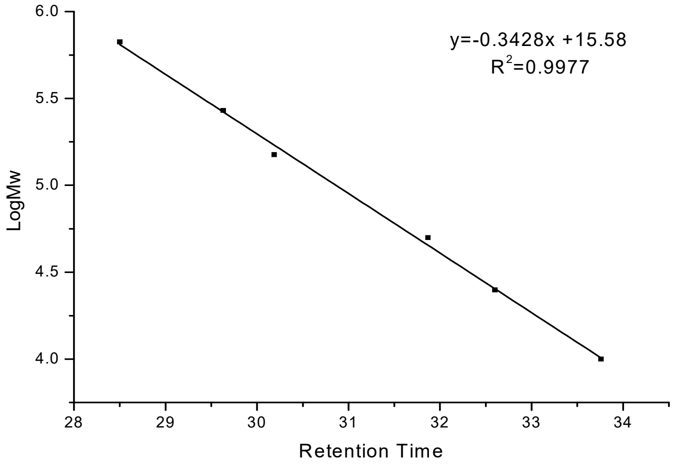

Figure S3. Standard curve of Dextrans.

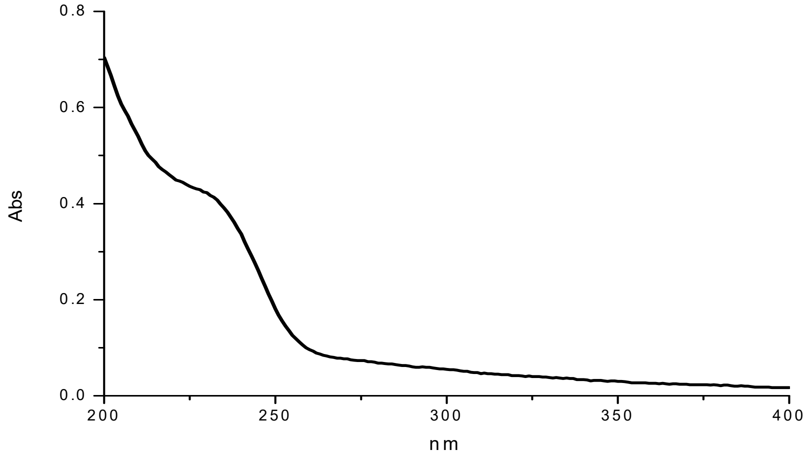

Figure S4. The UV spectrum of PAP-3
